# Circular RNAs Sparkle in the Diagnosis and Theranostics of Hepatocellular Carcinoma

**DOI:** 10.3389/fgene.2020.628655

**Published:** 2021-02-18

**Authors:** Menglan Wang, Minjie Wu, Tian Xie, Jianxiang Chen

**Affiliations:** ^1^College of Pharmacy, School of Medicine, Department of Hepatology, Institute of Hepatology and Metabolic Diseases, Institute of Integrated Chinese and Western Medicine for Oncology, The Affiliated Hospital of Hangzhou Normal University, Key Laboratory of Elemene Class Anti-Cancer Chinese Medicines, Engineering Laboratory of Development and Application of Traditional Chinese Medicines, Collaborative Innovation Center of Traditional Chinese Medicines of Zhejiang Province, Hangzhou Normal University, Hangzhou, China; ^2^Laboratory of Cancer Genomics, Division of Cellular and Molecular Research, National Cancer Centre Singapore, Singapore, Singapore

**Keywords:** HCC, circRNA, hepatocellular carcinoma, biomarker, therapy

## Abstract

Exonic circular RNAs (circRNAs) are a novel subgroup of non-coding RNAs, which are generated by a back-splicing mechanism of the exons or introns. Unlike the linear RNA, circRNA forms a covalently closed loop, and it normally appears more abundant than the linear products of its host gene. Due to the relatively high specificity and stability of circular RNAs in tissues and body fluid, circular RNAs have attracted widely scientific interest for its potential application in cancer diagnosis and as a guide for preclinical therapy, especially for hard-to-treat cancers with high heterogeneity, such as hepatocellular carcinoma (HCC). Thus, we summarize the updated knowledge of circular RNAs, including the mechanism of the generation of endogenous circular RNAs and their regulatory, diagnostic, and therapeutic roles in HCC.

## Introduction

The regulation of the mammalian transcriptome is diverse and complex. In humans, only 2% of the human genome is transcribed into protein-coding RNAs, and approximately 95% transcripts are non-coding mRNAs (Esteller, 2011; Han et al., 2018). As early as the 1970s, it has been found that circular RNAs (circRNAs) exist in viruses and eukaryotic genome (Kolakofsky, 1976; Sanger et al., 1976; Hsu and Coca-Prados, 1979). Due to technical limitation, few circRNAs were identified at that time. In recent years, along with the advancement in the technology of deep sequencing, plenty of circRNAs have been identified in eukaryotes (Jeck et al., 2013; Salzman et al., 2013; Szabo et al., 2016; Li J. et al., 2020). Accumulating evidence indicates that circRNAs are involved in a series of physiological and pathological pathways, resulting in many diseases, notably in cancers. Hepatocellular carcinoma (HCC) is a malignant cancer affecting people’s health globally. Due to the lack of a neural system in the liver, lesion of the liver in early stages is hard to be sensed because of a lack of symptoms. Mostly, patients with symptoms, like chest stuffiness and pain, are diagnosed at advanced stages with metastasis. The treatment outcome of late-stage HCC is dismal (Bray et al., 2018). The rapid lethality of late-stage HCC highlights the urgent need of early diagnosis and intervention (Zhou et al., 2018). CircRNAs consist of circular loops, which make them more stable and can be used as diagnostic markers (Qian et al., 2018). Due to the high abundance and stability and its unique expression signatures associated with cancer progression and prognosis, the potential of circRNAs acting as diagnostic and therapeutic biomarkers in HCC has been highlighted (Satoh and Yamamura, 2004; Hentze and Preiss, 2013; Filippenkov et al., 2017). In this review, we summarize the regulation of endogenous biosynthesis of circRNAs, their regulatory function and mechanisms in HCC, and the subsequent challenges and obstacles of applying circRNAs in clinical diagnosis and therapy.

## Endogenous Biosynthesis of Circrnas

**FIGURE 1:**
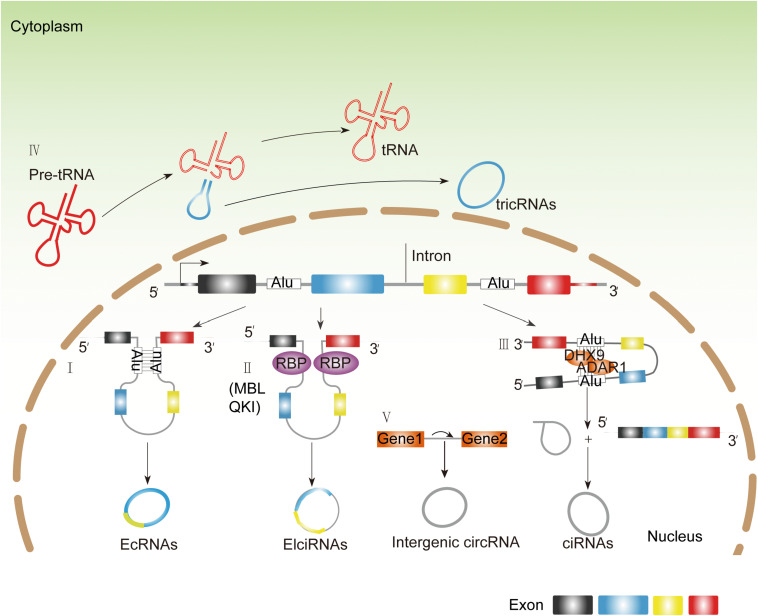
The endogenous biosynthesis of circRNAs. **(I)** Longflanking introns and inverted repeat elements (such as Alu elements) promote competition between the linear splicing and back-splicing of exons, leading to the generation of EcRNAs. **(II)** Some RBPs like MBL and QKI can recognize the specific motifs within flanking introns and directly bind on them, dragging two splicing sites close enough to promote back-splicing subsequently. **(III)** Some RBPs like DHX and ADAR1 disrupt base pairing between inverted repeat elements, allowing the splicing machinery to generate linear mRNA. **(IV)** TricRNAs are generated from introns spliced from pre-tRNA **(V)**. Intergenic circRNAs contains two intronic circRNA fragments.

The mechanism of production of circRNAs remains elusive. The most widely accepted model is that circRNAs are derived by a back-splicing mechanism. Based on the diversity of splicing sequences, circRNAs are categorized into four types: exonic circRNAs (ecircRNAs), exon–intron circRNAs (EIciRNAs), intronic circRNAs (including ciRNAs derived from pre-mRNAs and tricRNAs derived from tRNA intronic circular RNAs), and intergenic circRNAs (Figure 1; Jeck et al., 2013; Memczak et al., 2013; Salzman et al., 2013; Zhang et al., 2013; Ashwal-Fluss et al., 2014; Li Z. et al., 2015; Wang et al., 2016). Similar with the canonical splicing regulation, the formation of back-splicing requires the canonical splicing signals and spliceosome (Ashwal-Fluss et al., 2014; Andres-Leon et al., 2016). However, the established regulators and mechanism of back-splicing are still largely unknown. By far, RNA-binding proteins (RBPs) have been identified as the potential regulatory factors for *trans-*acting circRNA splicing. There are several RBPs that have been identified, such as Muscleblind (MBL) (Ashwal-Fluss et al., 2014), Quaking (QKI) (Conn et al., 2015), adenosine deaminase acting on RNA 1 (ADAR1) (Ivanov et al., 2015), and DExH-box helicase 9 (DHX9) (Aktas et al., 2017). As for MBL and QKI, they can recognize the specific motifs within flanking introns and directly bind on them, dragging two splicing sites close enough to promote back-splicing subsequently (Ashwal-Fluss et al., 2014; Conn et al., 2015). Conversely, some RNPs impede circRNA formation by destroying the pairing of intronic elements (Ivanov et al., 2015; Aktas et al., 2017). For example, ADAR1 inhibits circRNA formation by binding to double-stranded RNA and melting the stem structure, thus generating ecircRNAs and EIciRNAs, and ciRNAs are limited in the nucleus, indicating their variable functions (Zhang et al., 2013; Li Z. et al., 2015). The most common formulation of circRNAs is *via* “back-splicing.” There is a different way of RNA circularization occurring in archaea and eukaryotes, and circRNAs are derived from tRNA intron splicing (Figure 1; Noto et al., 2017).

## Regulatory Mechanisms of Circrnas on Biological Functions

CircRNAs are involved in physiogenesis and pathogenesis due to their complex biological functions. They exert cellular functions mainly by regulating transcription, alternative splicing (AS), translating into peptides, and acting as protein decoy or scaffold or miRNA sponges (Table 1). Their distinct biological functions are discussed below.

**TABLE 1:** Regulatory mechanisms of circRNAs on biological functions.

CircRNA	Model system	Function and mechanism
circMbl	Neuronal tissues	circMbl can compete with linear AS targets (Ashwal-Fluss et al., 2014).
circ-ZNF609	Myoblast	circ-ZNF609 has been confirmed with protein translation role in myogenesis by a splicing-dependent and cap-independent regulatory mechanism (Legnini et al., 2017).
circMbl3	ribosome footprinting from fly heads	the circMbl3-derived protein has been confirmed by mass spectrometry detection (Pamudurti et al., 2017).
circPABPN1	Cervical Carcinoma	circPABPN1 and PABPN1 mRNA bind to HuR competitively to suppress the translation of PABPN1(Abdelmohsen et al., 2017).
CircFoxo3	Non-cancer cells	CircFoxo3 inhibits the function of CDK2 via direct interaction with both CDK2 and p21 to form a ternary complex (Du et al., 2016).
circAmotl1	neonatal human cardiac tissue	The interaction of circAmotl1 with AKT1, STAT3, c-myc and PDK1 alters their localization translocating from cytoplasm to nucleus, which further regulates their downstream targets expression (Yang Q. et al., 2017; Zeng et al., 2017).
circZKSCAN1	HCC	circZKSCAN1 negatively regulates cancer stem cells by physically binding FMRP against CCAR1 complex (Zhu et al., 2019).
circSLC8A1	Bladder Cancer Cardiac Hypertrophy	circSLC8A1 acts as a sponge for miR130b/484 in bladder cancer (Lim et al., 2019) and a sponge of miR-133 in cardiac hypertrophy (Lu et al., 2019).

### CircRNAs Regulate Transcription and Alternative Splicing

**FIGURE 2:**
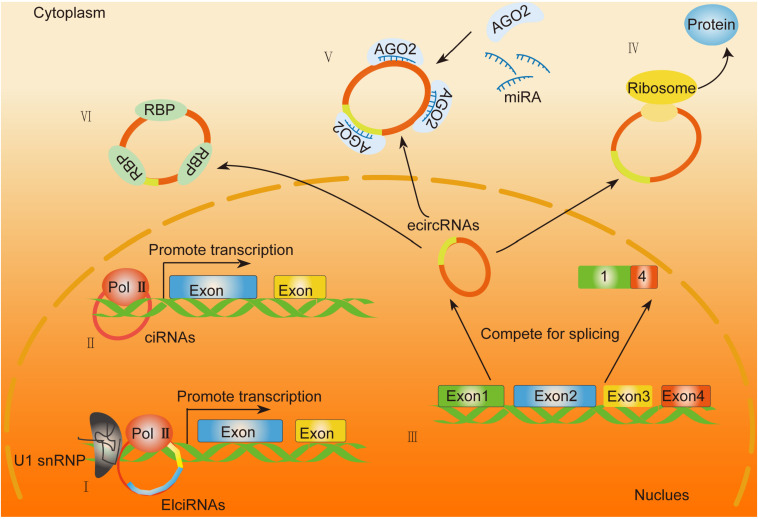
General biological functions of circRNAs. **(I,II)** EIciRNAs and ciRNAs function as enhancers of specific proteins by binding with the RNA polymerase II (Pol II) complex. **(III)** EcircRNAs are generated through back-splicing and translocate from the nucleus into the cytoplasm. **(IV)** Exonic circRNAs that contain internal ribosome entry sites (IRES) or prokaryotic binding sites translate into peptides. **(V)** EcircRNAs function as miRNA sponges. **(VI)** EcircRNAs can act as a decoy or scaffold to sequester proteins and regulate gene expression or functional protein localization.

As for different circRNAs, their locations in cells are quite different. EcircRNAs mainly exists in the cytoplasm, whereas EIciRNAs and ciRNAs mainly localize in the nucleus. EIciRNAs usually binds with U1 small nuclear ribonucleoprotein (U1 snRNP) *via* RNA–RNA interactions. The resultant complex further interacts with RNA polymerase II to enhance the transcription of ecircRNAs parental genes (Figure 2; Li Z. et al., 2015). Moreover, some circRNAs can compete with linear AS targets. For instance, circMbl competes with MBL pre-mRNA splicing during its formation (Ashwal-Fluss et al., 2014). Some circRNAs compete with linear splicing during transcription, leading to the production of circRNAs and linear mRNAs. Logically, due to the unfavorable assembly of spliceosomes at back-splicing sites, the efficiency of back-splicing is lower than canonical splicing. However, due to the damage of the core, pre-mRNA processing components like splicing factor 3A subunit 1 (SF3a1) and splicing factor 3B subunit 1 (SF3b1) are inhibited, leading to inhibited pre-mRNA splicing and enhanced back-splicing (Liang et al., 2017). Hence, the oscillation of circular and canonical AS could be a regulatory target for circRNA-mediated pathological activities (Zhang Y. et al., 2016; Liang et al., 2017; Vadlamudi et al., 2020).

### CircRNAs Translate Into Peptides

Although circRNAs are categorized into non-coding RNAs, emerging evidence has indicated the potential role of circRNAs in protein translation (van Heesch et al., 2019). Through ribosomal profiling, exonic circRNAs that contain internal ribosome entry sites (IRES) or prokaryotic binding sites have been proven with potential protein-coding capacity. Moreover, bioinformatics analysis has been involved in the identification of the open reading frame (ORF) and the potential IRES on the sequence of circRNAs. It has been reported that the translation capability of circRNAs mainly depends on the IRES element or m6A modification recently discovered (Yang Y. et al., 2017; Figure 2). For instance, circ-ZNF609 (Legnini et al., 2017) has been confirmed with a protein translation role in myogenesis by a splicing-dependent and cap-independent regulatory mechanism (Legnini et al., 2017). Moreover, the circMbl3-derived protein has been confirmed by mass spectrometry detection (Pamudurti et al., 2017). All the evidence above indicated a translational potential of circRNAs, which requires further exploration on their detailed regulatory mechanisms and their correlation with human diseases.

### CircRNAs Act as Protein Decoy or Scaffold

It has been reported that circRNAs can act as a decoy or scaffold to sequester proteins and regulate gene expression or functional protein localization (Figure 2). For example, circPABPN1 and PABPN1 mRNA bind to HuR competitively to suppress the translation of PABPN1 (Abdelmohsen et al., 2017). CircFoxo3 inhibits the function of CDK2 *via* direct interaction with both CDK2 and p21 to form a ternary complex (Du et al., 2016). The interaction of circAmotl1 with AKT1 (Zeng et al., 2017), STAT3 (Yang Q. et al., 2017), c-myc (Yang Q. et al., 2017), and PDK1 (Zeng et al., 2017) alters their localization translocating from the cytoplasm to the nucleus, which further regulates their downstream target expression. In HCC, some RBP is involved in HCC progression. For instance, circZKSCAN1 negatively regulates cancer stem cells by physically binding FMRP against the CCAR1 complex in HCC. It exerts its inhibitive role by competitively binding with FMRP, therefore blocking the binding of FMRP and β-catenin-binding protein-cell cycle and apoptosis regulator 1 (CCAR1) mRNA and subsequently restraining the transcriptional activity of Wnt signaling (Zhu et al., 2019). Conclusively, the capability of circRNAs to interact with proteins may mainly depend on the nucleotide sequences and the secondary or tertiary structures of each circRNA, the detailed mechanism of which needs further precise study.

### CircRNAs Function as MiRNA Sponges

To date, numerous reports have elucidated a common function of circRNAs as miRNA sponges. A large number of circRNAs localize in the cytoplasm, indicating their role in posttranscriptional regulation. The function of circRNAs as miRNA sponges was first identified in 2013, and the human circRNA running antisense to the Cerebellar Degeneration-Related protein 1 (CDR1) locus, termed as CDR1as, harbors about 70 conserved matches to the miR-7 seeds (Memczak et al., 2013). CDR1as also binds with AGO protein to enhance the expression of miR-7 targets (Hansen et al., 2013). Some circRNA serves as sponges for different miRNAs. For instance, circSLC8A1 acts as a sponge for miR130b/484 in bladder cancer (Lim et al., 2019) and a sponge of miR-133 in cardiac hypertrophy (Lu et al., 2019).

## The Role of Circrnas in Human Hepatocellular Carcinoma

By far, numerous evidences have suggested that dysregulation of circRNAs is closely correlated with cancer initiation and/or progression. According to their roles in HCC progression, they can be classified into oncogenes, tumor suppressors, and circRNAs that interfere with hepatitis virus infection (Table 2). The other cancer-related roles of circRNAs were recently indicated in drug resistance and heterogeneity [Roles of circRNAs in the tumor microenvironment, Molecular Cancer volume 19, Article number: 14 (2020)], which is remarkable and widely detected in human HCC. Thus, we aim to summarize the circRNAs recently reported in the regulation of HCC. Due to space limitations, we apologize to the authors for the literature not listed and discussed here.

**TABLE 2:** Representative circRNAs in the regulation of HCC.

CircRNA	Function	Mechanism
circRNA_100338	Oncogene	Function as a sponge of smiR-141-3p (Huang et al., 2017).
circ_0005075	Oncogene	Function as a sponge of miR-431 (Li et al., 2018).
circ-ZNF652	Oncogene	Function as a sponge of miR-29a-3p/GUCD1 (Li Y. et al., 2020).
circMAST1	Oncogene	Function as a sponge of miRNA-1299 Stabilizing CTNND1 (Yu X. et al., 2020).
circZNF609	Oncogene	Function as a sponge of miR-15a-5p/15b-5p Activating the Hedgehog pathway (He et al., 2020).
exosome circ-deubiquitination (circ-DB)	Oncogene	Function as a sponge of miR-34a Activating deubiquitination-related USP7 (Zhang et al., 2019).
circ_0091579	Oncogene	ND (Jiang et al., 2020)
hsa_circ_0000711	Oncogene	Targeting has-miR-103a-3p (Chen K. H. et al., 2020).
Hsa_circ_104566	Oncogene	Decreasing apoptosis and E-cadherin (Liu et al., 2020).
circRNA-100338	Oncogene	Enhancing invasiveness and angiogenesis (Huang et al., 2020).
hsa_circ_0000092	Oncogene	hsa_circ_0000092 competitively bind to miR-338-3p to up-regulate HN1 expression (Pu et al., 2020).
ciRS-7 (Cdr1as)	Oncogene	ND (Xu et al., 2017).
circZKSCAN1	Tumor suppressor	Inhibits Wnt signaling (Zhu et al., 2019).
circ-MTO1	Tumor suppressor	Function as a sponge of miR-9 Promote p21 expression (Han et al., 2017).
cSMARCA5	Tumor suppressor	Function as a sponge of miR-17-3p Function as a sponge of miR-181b-5p (Yu et al., 2018).
circ- 102,166	Tumor suppressor	Function as a sponge of miR-182 Function as a sponge of miR-184 (Li R. et al., 2020).
circRNA-ITCH	Tumor suppressor	Regulating Wnt/ββ-catenin signal transduction (Yang et al., 2020).
circ-0051443	Tumor suppressor	Promoting cell apoptosis and arresting the cell cycle (Chen W. et al., 2020).
circ_4911	Tumor suppressor	Inhibits the formation of human umbilical vein endothelial cells (HUVECs) (Yan et al., 2020).
circ_4302	Tumor suppressor	Inhibits the formation of human umbilical vein endothelial cells (HUVECs) (Yan et al., 2020).

### CircRNAs Function as Oncogenes in HCC

There have been numerous circRNAs identified in HCC, related with the promotion of cancer cells’ proliferation and invasion, inhibition of apoptosis, and enhancement of angiogenesis. For instance, circRNA_100338 functions as a sponge of miR-141-3p to promote invasion of HCC cells. The expression of circRNA_100338 is positively correlated with poorer of OS and PFS of HCC patients (Huang et al., 2017). circ_0005075 promotes HCC progression *via* miR-431 (Li et al., 2018), and exosomal circ-ZNF652 could transfer to HCC cells to promote cell proliferation, migration, invasion, and glycolysis in HCC *via* the miR-29a-3p/GUCD1 axis (Li Y. et al., 2020). CircMAST1 elicits HCC progression by sponging miRNA-1299 and stabilizing CTNND1. CircMAST1 is upregulated in HCC tissues and cell lines; silencing circMAST1 with small interfering RNA inhibits the migration, invasion, and proliferation of HCC (Yu X. et al., 2020). CircZNF609 enhances HCC cell proliferation, metastasis, and stemness by activating the Hedgehog pathway through the regulation of miR-15a-5p/15b-5p and GLI2 expressions (He et al., 2020). Exosome circ-deubiquitination (circ-DB) is upregulated in HCC patients with higher body fat ratios. Exo-circ-DB promotes HCC growth and reduces DNA damage *via* suppressing miR-34a expression and activating deubiquitination-related USP7 (Zhang et al., 2019). Circ_0091579 promotes HCC progression by enhancing cell migration and invasion and impeding cell apoptosis (Jiang et al., 2020). hsa_circ_0000711 promotes proliferation and inhibits the apoptosis of hepatoma cells *via* targeting has-miR-103a-3p (Chen K. H. et al., 2020). Hsa_circ_104566 promotes HCC progression by decreasing apoptosis and E-cadherin, thus increasing cell viability, proliferation, migration, invasion, and N-cadherin (Liu et al., 2020). Exosomal circRNA-100338 promotes HCC metastasis by enhancing invasiveness and angiogenesis (Huang et al., 2020). hsa_circ_0000092 competitively binds to miR-338-3p to upregulate HN1 expression, promoting angiogenesis in HCC (Pu et al., 2020). Circular RNA ciRS-7 (Cdr1as) acts as a risk factor of hepatic microvascular invasion in HCC (Xu et al., 2017). This evidence suggested that circRNAs play important roles in HCC progression *via* regulating angiogenesis.

### CircRNAs Act as Tumor Suppressors in HCC

Conversely, there are some circRNAs that have been identified as tumor suppressors. circZKSCAN1 inhibits Wnt signaling to impede cancer stemness property and malignancy in HCC (Zhu et al., 2019). circ-MTO1 suppresses HCC progression by acting as a sponge of oncogenic miR-9 to promote p21 expression (Han et al., 2017). Expression of circ-MTO1 is negatively correlated with the prognosis of HCC patients (Han et al., 2017). cSMARCA5 inhibits the growth and migration of HCC by sponging of miR-17-3p and miR-181b-5p to promote the expression of TIMP3, a well-known tumor suppressor (Yu et al., 2018). Circular RNA circ-102,166 acts as a sponge of miR-182 and miR-184 to suppress HCC proliferation and invasion (Li R. et al., 2020). CircRNA-ITCH inhibits cell proliferation and promotes apoptosis through regulating Wnt/β-catenin signal transduction, preventing the occurrence of HCC (Yang et al., 2020). Circ-0051443, an exosomal circRNA, can transmit from normal cells to HCC cells *via* exosomes and suppress the malignant biological behaviors by promoting cell apoptosis and the cell cycle arrest (Chen W. et al., 2020). HCC is a typical type of cancer that is hypervascular. Endothelial cells have been confirmed to participate in angiogenesis and influence the development of HCC (Heimann et al., 1996). Circ_4911 and circ_4302 inhibits the formation of human umbilical vein endothelial cells (HUVECs) in the microenvironment of HCC (Yan et al., 2020). All these circRNAs have proven to have the potential to be therapeutic targets for HCC (Table 1). However, it is necessary to solve the limitation of the strategies that target them.

### CircRNAs Interfere With Hepatitis Virus Infection in HCC Progression

Hepatitis virus infection is the primary cause of HCC. Chronic hepatitis B virus (HVB) infection takes the dominant risk factor in the majority of the areas of Asia and sub-Saharan Africa that have a high incidence of HCC (Cui et al., 2018). The group of Ding has screened the circRNAs in HVB-related HCC patients by microarray. They have revealed 24 upregulated and 23 downregulated circRNAs significantly (fold-change ≥ 2; *P* ≤ 0.05) in HCC tissues compared with non-tumorous tissues (NTs) (Cui et al., 2018). The top five upregulated circRNAs are hsa_circRNA_104351, hsa_circRNA_102814, hsa_circRNA_103489, hsa_circRNA_102109, and hsa_circ RNA_100381, and the top five downregulated circRNAs are hsa_circRNA_100327, hsa_circRNA_101764, hsa_circ RNA_101092, hsa_circRNA_001225, and hsa_ circRNA_102904 (Cui et al., 2018). Among them, cRNA_101764 may play a dominant role in suppressing HCC progression through the PI3K–Akt signaling pathway (Wang et al., 2018).

## Translational Potential of Circrna

### CircRNAs Acting as Biomarkers

The expression of circRNAs exhibits dynamic global changes during development (Jiao et al., 2020). For example, in humans, induction is observed across a variety of tissues and is consistently observed for circRNAs spliced by both the major (U2) and minor (U12) spliceosome (Barrett and Salzman, 2016). This may be an induction that circRNAs exhibit a high degree of tissue specificity, and some correlates with the size of tumor, with the TNM stage, or metastasis (Zheng et al., 2019). Due to this specificity of circRNAs, they can be used as biomarkers for early cancer detection. Early detection of cancers has been a promising research focus globally. In the past years, circRNAs acting as biomarkers have attracted increasing interest for early detection of cancer. Several characteristics of circRNAs indicate the advantages of circRNAs as biomarkers. (a) Specific expression and high conservation: CircRNAs are expressed in a tissue/development stage-specific manner and most of them are conserved in variable species (Guo et al., 2014; Jeck and Sharpless, 2014; Wang et al., 2014; Zhang Y. G. et al., 2016); (b) stable structure: circRNAs are covalently closed loops, lacking of 5^–^–3^–^ polarity and polyadenylated tails. Thus, circRNAs are resistant to RNase or RNA exonuclease activation (Zhang Y. G. et al., 2016), due to their higher stable structures compared with the linear RNAs (Suzuki and Tsukahara, 2014); and (c) high abundance: the abundance of circRNAs is comparable with its canonical linear transcripts (Salzman et al., 2012). CircRNAs can be detected in both tumor tissues and body fluids, including blood, saliva, and urine (Bahn et al., 2015; Li Y. et al., 2015; Vo et al., 2019). The specific circRNAs detected in body fluids would be useful indicators of cancer or diseases (Sand et al., 2016), which makes circRNAs ideal biomarkers for invasive detection.

### Potential of CircRNAs in HCC Therapy

Although there are numerous reports of the functions of circRNAs in HCC, it is disappointing that there is no application of circRNAs in clinical cancer therapy directly. Only few of the researchers have tested and evaluated the potential application of targeting circRNAs in preclinical animal models. CircMYLK was determined to be significantly upregulated in HCC tissues and cells, and mouse tumorigenicity assay shows that injection of circMYLK small interfering RNA (siRNA) drastically suppresses xenograft tumor formation *in vivo* (Gao et al., 2020). Silencing circMAST1 with siRNA inhibits xenograft tumor migration, invasion, and proliferation in mouse (Yu X. et al., 2020). Recently, a group established a plasma circular RNA panel to diagnose hepatitis B virus-related HCC (Yu J. et al., 2020). Three plasma circRNAs are identified, namely hsa_circ_0000976, hsa_circ_0007750, and hsa_circ_0139897, which show higher accuracy than the serum biomarker alpha-fetoprotein (AFP) (Yu J. et al., 2020). This provides a guidance for HCC detection with serum circRNA. However, there are some limitations for this circpanel; for instance, all the HCC patients in this study were HBV-related, and further study of HCC caused by other factors should be evaluated. The application of circRNA in clinical treatment still needs a long way for development in the future.

## Translational Challenges and Perspectives of Circrnas in HCC

CircRNAs have shown great potential as biomarkers for early cancer detection and as targets of cancer therapy (Fu et al., 2020; Sun et al., 2020; Zhu et al., 2021); however, some obstacles still need to be overcome. Firstly, the clinical relevance of circRNAs toward given cancers needs more mechanistic investigation and correlation analysis with a large cohort of patient samples to be confirmed. Secondly, technical improvement on the quantification of a specific circRNA and silencing it without affecting the expression of the parental linear transcript are in urgent need. Thirdly, as the detection hairpin probes should be designed to the back-splice junction sites, the clinical application of circRNAs is limited due to the lack of specific capture and detection in biopsy.

Recently, some research groups have established methods for the accurate quantification of circRNAs with an algorithm, CIRIquant, a consolidated computational pipeline, which helps unveil the regulation of competitive splicing between circRNAs and their linear counterparts (Wang et al., 2018; Di Liddo et al., 2019; Zhang et al., 2020). Zheng et al. have identified a new feature, reverse overlap (RO), for circRNA detection, which outperforms back-splice junction (BSJ)-based methods in identifying low-abundance circRNAs (Wang et al., 2018). Electrochemical detection of circRNAs combines back-splice junction recognition and duplex-specific nuclease-assisted target recycling signal amplification (Castañeda et al., 2017; Jiao et al., 2020). Due to its high sensitivity and reproducibility, it has been employed to assay circRNA in different concentrations into human 1% serum, 10% serum, and 10% peripheral blood to test the repeatability and stability of this method (Castañeda et al., 2017; Jiao et al., 2020). Even though these methods have shown excellent sensitivity, repeatability, and stability in experiments, but how about when these methods are applied in clinical detection? An experiment with large cohorts of HCC patients is needed for the evaluation of circRNA detection with these methodologies.

Moreover, the application of precise RNA interference (RNAi) to target oncogenic circRNAs in cancers should exclude the possibility of interference with the expression of cognate linear mRNAs. As for this purpose, the interference RNAs should be designed to accurately target the unique back-spliced junctions of oncogenic circRNAs. HCC is one of the most malignant cancers worldwide. There is a great need for the combination of interdisciplines to develop clinical tools for early detection and therapy of HCC. In the past few years, a plethora of studies have revealed that circRNAs are systematically altered in HCC. The characterization of the mechanisms by which these circRNAs contribute to cancer offers opportunities for the early diagnosis, evaluation of prognosis, and therapeutic intervention of HCC. There are lots of circRNAs identified to be involved in HCC tumor cell-autonomous processes, including cell proliferation, apoptosis, invasion, and metastasis, but the functions of circRNAs in the tumor microenvironment of HCC are limited (Chen Y. et al., 2020; Zhou et al., 2020), which needs further investigation. To date, most researches on circRNAs are identified based on HCC cell lines. It is worth noting that HCC has high heterogeneity, and it is not precise to draw a functional conclusion about a specific circRNA studied within limited HCC cell lines. Thus, it is urgent to develop tools to detect circRNAs with body fluid of patients. Although there is a long way to go for the clinical translation of circRNAs, the rapid advancement of technologies and increasing research in the area of circRNA will make the “dark world” of circRNAs enlightened.

## Author Contributions

JC and TX designed and instructed the structure of this manuscript. MeW and MiW wrote this manuscript and make the revisions.

## Conflict of Interest

The authors declare that the research was conducted in the absence of any commercial or financial relationships that could be construed as a potential conflict of interest.
